# Immune dysregulation, apoptosis impairment, and enhanced seroreactivity to *Anisakis simplex* in Crohn’s disease: interplay of IL-7/IL-7R signalling and CD132 deficiency

**DOI:** 10.1590/0074-02760250129

**Published:** 2026-04-10

**Authors:** Carmen Cuéllar, Carolina Hurtado-Marcos, Elizabeth Valdivieso, Lucianna Vaccaro, Juan González-Fernández, Ana Isabel Jiménez, Jaume Pérez-Griera, Salvador Benlloch, Cirilo Amorós, Rafael Gil-Borrás, Rosa Sorando-Serra, María José Cano-Cano, Juan Carlos Andreu-Ballester

**Affiliations:** 1 Complutense University of Madrid, Department of Microbiology and Parasitology, Madrid, Spain; 2 Complutense University of Madrid, Parasitic Immunobiology and Immunomodulation Research Group, Madrid, Spain; 3 CEU Universities, Universidad CEU San Pablo, Urbanización Montepríncipe, Boadilla del Monte, Spain; 4 Arnau de Vilanova Hospital, Biopathology Department, Valencia, Spain; 5 University Clinical Hospital, Laboratory Department, Valencia, Spain; 6 Arnau de Vilanova Hospital, Digestive Department, Valencia, Spain; 7 CEU Universities, Cardenal Herrera, Valencia, Spain; 8 Arnau de Vilanova Hospital, Emergency Department, Valencia, Spain; 9 Foundation for the Promotion of Health and Biomedical Research in the Valencian Region, Benimaclet, Valencia, Spain

**Keywords:** Crohn’s disease, anti-*Anisakis simplex* antibodies, IL-7 gene expression, IL-2 receptor subunit g (CD 132), caspase-3

## Abstract

**BACKGROUND:**

In previous studies, we identified a deficiency of γδ T cells and an increased prevalence of anti-*Anisakis simplex* antibodies in patients with Crohn’s disease (CD). Additionally, decreased gene expression of the interleukin 2 (IL-2) receptor γ subunit (CD132) was observed in tissues from CD patients.

**OBJECTIVE:**

To analyse the gene expression of IL-7 and its receptors in tissues from CD patients and to explore its relationship with anti-*A. simplex* antibodies.

**METHODS:**

52 patients diagnosed with CD were compared with a control group of 52 healthy individuals. Peripheral blood samples were analysed to assess levels of anti-*A. simplex* antibodies and IL-7. In addition, intestinal tissue samples from 20 subjects in each group were examined to evaluate IL-7 gene expression, IL-7 protein levels, the IL-2 receptor γ subunit (CD132), the IL-7 receptor α subunit (CD127), and caspase-3 expression.

**FINDINGS:**

Anti-*A. simplex* antibody levels were elevated in patients with CD. Caspase-3 expression was significantly reduced in the tissues of CD patients with anti-*A. simplex* IgA, and this reduction extended to IgG and IgE in healthy individuals. A negative correlation was observed between caspase-3 levels and serum anti-*A. simplex* IgA, as well as IL-7 levels in the tissues of CD patients. In healthy subjects, tissue IL-7 levels were lower in those positive for anti-*A. simplex* IgA, while serum IL-7 levels were higher in individuals positive for anti-*A. simplex* IgG.

**MAIN CONCLUSIONS:**

This study revealed the interplay between IL-7 signalling, γδ T cell deficiency, and immune responses to *A. simplex* in CD. Our findings underscored a cause-effect relationship between CD132 deficiency, γδ T cell depletion, and defective mucosal immunity, which may drive both CD inflammation and susceptibility to parasitic infections like *A. simplex*.

## INTRODUCTION

The aetiology of Crohn’s disease (CD), a chronic inflammatory disorder of the gastrointestinal tract, remains elusive. However, its pathophysiology is believed to involve alterations in the innate immune system, particularly in genetically susceptible individuals exposed to specific pathogens.^1^ Dysregulation of the innate immune system has been reported, including deficiencies in γδ T cells, which are predominantly located in mucosal membranes and serve as a critical first line of defence against pathogenic threats.^2^

The disease has also been linked to dysbiosis within the gut microbiota, characterised by an imbalance between beneficial microbes and pathogenic species. Emerging evidence highlights the role of newly identified intestinal microbes with pathogenic properties, termed “pathobionts,” in the development and progression of CD. These pathobionts exhibit unique mechanisms that contribute to chronic inflammation, including escape of immune regulation and exacerbation of intestinal damage.^3^

The relationship between intestinal anisakiasis and CD has been explored in several studies.^4^ ^5^ ^6^ ^7^ *Anisakis simplex*, a nematode parasite, uses crustaceans and fish as intermediate hosts, with humans serving as accidental hosts. Human anisakiasis occurs upon ingestion of these larvae, leading to gastrointestinal and systemic infections often accompanied by allergic reactions, representing a significant public health concern.^8^

Studies have identified an association between specific anti-*A. simplex* antibodies and a deficiency in γδ T cells, suggesting that these cells play a critical role in the immune response against the parasite.^9^ Recent research has demonstrated that patients with CD exhibit deficient gene expression of interleukin 2 (IL-2) receptor subunit γ (CD132), a component of the IL-7 receptor essential for γδ T cell stimulation and proliferation.^10^ Furthermore, CD patients show reduced γδ T cell levels in tissues and peripheral blood, increased apoptosis of these cells, and decreased caspase-3 activity in the affected tissues. These findings highlight the potential immunological interplay between anisakiasis and the pathogenesis of CD. Taken together, these findings highlight the clinical relevance of IL-7R modulation in CD. The altered IL-7/IL-7R signalling pathway we describe, influenced by *A. simplex* immune responses, may contribute not only to defective γδ T cell homeostasis but also to disease persistence and progression. Since IL-7R expression (particularly the common γ-chain, CD132) has been proposed as a potential biomarker of treatment resistance and disease severity in CD,^11^ our data suggest that anti-*A. simplex* antibodies and IL-7R dysfunction could serve as combined indicators of mucosal immune dysregulation. Moreover, therapeutic strategies aimed at restoring IL-7R signalling or mimicking the immunomodulatory effects observed in parasite exposure could open novel avenues for diagnosis and treatment in CD.

*Anisakis simplex* is of specific interest in CD due to overlapping epidemiological, clinical, and immunological features that suggest a possible role for this parasite in CD pathogenesis and presentation. Epidemiologically, anti-*Anisakis* antibodies have been reported at higher prevalence among CD patients than in healthy controls, with some studies showing specific immunoglobulins (notably IgA and IgG) detected in up to 29-44% of CD patients, which is disproportionately high compared to the healthy population.^5^ ^12^

Clinically, intestinal anisakiasis and CD share overlapping symptoms such as abdominal pain and granulomatous inflammation, and *Anisakis* infection can mimic the presentation of CD, occasionally leading to diagnostic confusion and unnecessary interventions. There are documented cases in which *Anisakis* infection was initially misdiagnosed as CD based on clinical and histopathological findings.^12^

Immunologically, *A. simplex* infection stimulates a pronounced Th2-type immune response with increased levels of specific IgE, IgA, and IgG antibodies, as well as local eosinophilia, parameters that coincide with the immunological profile often observed in CD. In CD patients, the presence of anti-*Anisakis* IgA has been associated with higher CD activity indices, supporting a possible modulatory or exacerbating influence of the parasite’s antigens on disease severity and mucosal immune activation.^5^ ^12^

In summary, *A. simplex* represents a frequent, clinically relevant, and immunologically active coinfection in patients with CD, justifying its particular interest in the context of CD pathogenesis, diagnosis, and the broader understanding of host-parasite interactions in inflammatory bowel conditions.^5^ ^12^

Our objective was to analyse the gene expression of IL-7 and its receptors in the tissues of patients with CD, with the aim of establishing a relationship between this cytokine and anti-*A. simplex* antibodies.

## SUBJECTS AND METHODS

### Study population

We conducted a prospective case-control study to analyse the peripheral blood of 104 individuals, including 52 patients diagnosed with CD and 52 healthy controls. Additionally, we compared the tissue samples from 20 of these patients with those from 20 healthy subjects.

Intestinal tissue samples from CD patients were obtained via endoscopic or surgical biopsies, whereas control tissues from healthy subjects were collected during protocolised colon cancer screening programs showing normal findings. Participants were recruited at Arnau de Vilanova Hospital (Valencia, Spain), and patients and healthy controls were matched by sex and age (± 5 years). CD diagnosis adhered to the Lennard-Jones criteria, which integrate clinical, endoscopic, radiological, and histopathological features, including transmural inflammation, granulomas, and mucosal discontinuity.^13^ Disease activity was assessed using the CD Activity Index (CDAI), calculated from weighted parameters, such as stool frequency, abdominal pain, and haematocrit levels.^14^ The patients were stratified into three groups:

Newly diagnosed: active CD at presentation with no prior treatment or treatment initiation within ≤ 24 h.

Remission: sustained CDAI < 150 for ≥ 12 months.

Active disease: CDAI > 150.

Healthy controls excluded individuals with recent vaccinations (within three months), immunosuppressive therapies, immunodeficiencies, or autoimmune/inflammatory conditions.

### Tissue sampling of intestinal biopsies

Three to five biopsy specimens from the ileum and colon were collected at 1-2 cm intervals in each subject. Select samples underwent fixation via immersion in 10% neutral-buffered formalin solution (pH 7.4) and were routinely processed through paraffin embedding for histological analysis. Tissue sections (4-5 μm thickness) were mounted on glass slides and stained with haematoxylin-eosin for microscopic evaluation.

Parallel samples were snap-frozen in liquid nitrogen without prior fixation to preserve the macromolecular integrity for subsequent cellular and molecular profiling. Frozen specimens underwent mechanical homogenisation in a dissociation medium containing phosphate-buffered saline (PBS, pH 7.4), 1% foetal bovine serum (FBS), 1 mM dithiothreitol (DTT), and 1 mM ethylenediaminetetraacetic acid (EDTA), followed by incubation at 37ºC for 15 min. After centrifugation at 300 × g for 5 min, the cellular pellet was subjected to enzymatic digestion using 0.5 mg/mL collagenase type VIII (Sigma-Aldrich) in 5% FBS-supplemented medium, with continuous agitation at 37ºC for 30 min.

The resulting cell suspension was filtered through a 70-μm nylon mesh, pelleted by centrifugation, and immunolabeled with fluorochrome-conjugated antibodies targeting lineage-specific surface markers for flow cytometric analysis. Intestinal lymphocytes were isolated from healthy controls and CD patients for comparative functional studies.

### Determination of anti-*A. simplex* specific antibodies

Enzyme-linked immunosorbent assay (ELISA) plates (Costar, Corning, NY, USA) were coated with larval antigen at a concentration of 10 μg/mL. Human serum samples, diluted 1:100 in PBS-Tween containing 0.1% bovine serum albumin (BSA), were added and incubated. Detection was performed using horseradish peroxidase (HRP)-conjugated goat anti-human IgM, IgG, or IgA antibodies (BioSource International, Camarillo, CA, USA). For IgE determination, serum samples were diluted 1:2 and incubated with a murine monoclonal antibody specific for the epsilon chain of human IgE (IgG1κ, clone E21A11; INGENASA, Madrid, Spain). Subsequently, a goat anti-mouse IgG1 (γ) HRP conjugate (Life Technologies, Grand Island, NY, USA) was used.

The participants were categorised into two groups based on their anti-*A. simplex* antibody levels for quantitative comparisons. Positive results were defined as optical density (OD) values exceeding the mean OD of the studied sera plus two standard deviations for each immunoglobulin type.^9^

### IL-7 in peripheral blood

The concentration of serum interleukin-7 (IL-7) was quantified using an ELISA kit (Quantikine® HS ELISA, R&D Systems, Catalogue #: HS750) following the manufacturer’s protocol.

### Cell isolation for the analysis of γδ and αβ T lymphocytes and apoptosis assessment

Blood cell counts were performed using an automated haematology analyser (LH750; Beckman Coulter, Inc., Fullerton, CA, USA). To enrich the sample in mononuclear cells, density gradient centrifugation was performed on EDTA-anticoagulated blood using Lymphoprep™ (Palex Medical SA, Barcelona, Spain). Following two PBS washes, the collected cells were resuspended in 200 μL of binding buffer from the ANNEXIN V-FITC/7-AAD Kit (Beckman Coulter, Inc.), in the presence of calcium.

### Analysis of γδ and αβ T cells

To assess the functional profile of γδ and αβ T lymphocytes in peripheral blood and intestinal tissue, flow cytometry was performed using the following monoclonal antibodies: Anti-TCR PAN αβ-PE (Catalogue #: B49177), Anti-TCR PAN γδ-PE and FITC (Catalogue #: 6607015), CD19-PC7 (Catalogue #: IM3628), CD56-PC7 and PE (Catalogue #: A21692; B36214), CD4-PC7 (Catalogue #: 737660), CD3-PC5 and ECD (Catalogue #: A07749; 6607013), CD8-PC7 and FITC (Catalogue #: 6607013), CD5-FITC (Catalogue #: IM0468U), and CD45-ECD (Catalogue #: 6607013) (Beckman Coulter). A total of 100,000 events were acquired using a multiparameter Navios flow cytometer (Beckman Coulter), and data were subsequently analysed with Kaluza software.

### Apoptosis evaluation

Apoptosis in peripheral blood was assessed using the ANNEXIN V-FITC/7-AAD Kit (Beckman Coulter), which relies on Annexin V’s affinity for phosphatidylserine exposed on apoptotic cells and the specificity of 7-amino-actinomycin D (7-AAD) for DNA guanine-cytosine base pairs. The assay was performed according to the manufacturer’s instructions.

### Gene expression of IL-7, IL-7 receptor α (CD127), and IL-2 receptor γ subunit (CD132) in tissues. Reverse transcription real-time polymerase chain reaction (RT-qPCR)

Tissue samples were homogenised in 1 mL of TRIzol® Reagent (Ambion, Life Technologies, Carlsbad, CA, USA) for RNA isolation, following the manufacturer’s instructions. RNA concentration was determined using a GE NanoVue Spectrophotometer (GE Healthcare Life Sciences, Little Chalfont, UK).

Reverse transcription was performed using 1 μg of extracted RNA with a Thermo Scientific RevertAid H Minus First Strand cDNA Synthesis Kit (Thermo Fisher Scientific, Waltham, MA, USA. Catalogue Number K1621). The reaction was carried out by preparing the mixture with RNA template (1 μg), oligo dT (1 μL), and nuclease-free water up to 12 μL. Then, 5X Reaction Buffer (4 μL), RNase Inhibitor (20 U/μL, 1 μL), 10 mM dNTP mix (2 μL), RevertAid M-MuLV RT enzyme (200 U/μL, 1 μL), and water were added to a final volume of 20 μL. The mixture was incubated for 60 min at 42ºC, and the reaction was terminated by heating at 70ºC for 5 min. The product of the first strand cDNA synthesis can be used directly qPCR using SYBR Green Real Time PCR master mix Kit (Thermo Fisher Scientific, Waltham, MA, USA. Catalogue Number 4309153). Used to perform in a GeneAmp 5700 system (Applied Biosystems Foster City, CA, USA). For this, the reaction was carried out with 100 ng of cDNA from each sample, forward primer (100 nM, 1 μL), reverse primer (100 nM, 1 μL), 2X SYBR Green PCR Master Mix (25 μL), and water up to a final volume of 50 μL PCR conditions were as follows: 5 min at 94ºC; 40 cycles of 30 s at 94ºC, 30 s at 60ºC; 60 s at 72ºC. Glyceraldehyde 3-phosphate dehydrogenase (GAPDH) served as the internal reference gene. Gene expression level were calculated by the 2^-ΔΔCT^ method. Primer sequences are listed in Supplementary data (Table A).

### IL-7 and caspase-3 protein expression. Western-blot

Western blot analysis was performed using protein extracts from the intestinal biopsies. Protein purification was performed using the Trizol/Guanidine method with TRIzol® Reagent (Ambion, Life Technologies) and Guanidine Hydrochloride (SIGMA), following the manufacturer’s guidelines. The extracts were preserved in TNT buffer (20 mM Tris-HCl, pH 7.5, 0.2 M NaCl, 1% Triton X-100) and supplemented with a protease inhibitor cocktail (10%) (Roche, Vienna, Austria) immediately before use. Protein concentration was measured using the Pierce BCA Protein Assay Reagent (Thermo Fisher Scientific, Waltham, MA, USA).

A total of 20 μg of protein was separated by sodium dodecyl sulphate-polyacrylamide gel electrophoresis (SDS-PAGE) using 12% polyacrylamide gels. Proteins were subsequently transferred onto a nitrocellulose membrane using the Bio-Rad Mini Protean II system, following the manufacturer’s recommendations (Bio-Rad, Hamburg, Germany).

Membranes were incubated overnight at 4ºC with the corresponding primary antibody [anti-IL-7 or anti-caspase-3; Supplementary data (Table B)] in TBS buffer (20 mM Tris-HCl, pH 7.5, and 150 mM NaCl) containing a 0.1% blocking agent. Excess primary antibody was removed by washing with TBS supplemented with 0.05% Tween-20, followed by incubation with a 1:1000 dilution of secondary antibody (Merck, Darmstadt, Germany). After additional washes, positive bands were visualised using an image digitiser with enhanced chemiluminescence (ECL) reagent (Amersham, Little Chalfont, UK) and quantified by densitometry using ImageJ software. Actin was used as an internal loading control.

### Statistical analysis

The Mann-Whitney U test was employed to assess differences in medians between two independent groups of quantitative variables, as it is a robust non-parametric alternative to the t-test when data do not meet normality assumptions. Contingency tables were constructed to examine the relationship between anti-*A. simplex* antibodies, and other qualitative variables. For this purpose, chi-square tests and Fisher’s exact tests were used to determine statistical associations.

Spearman’s correlation coefficient was used for correlation analyses, comparing IL-7 gene expression, serum IL-7 levels, caspase-3 activity, and anti-*A. simplex* antibody levels. Graphical representations were generated using GraphPad Prism (version 8.0.0; GraphPad Software, San Diego, CA, USA). All statistical analyses were conducted using Statistical Package for Social Sciences (SPSS) version 19.0 (SPSS Inc., Chicago, IL, USA).

### Ethical approval

This research was conducted in accordance with the recommendations of the Spanish Bioethics Committee, the Spanish legislation on Biomedical Research (Law 14/2007), and the Personal Data Protection Law (Law 3/2018 and European Law UE676/2018). The study was reviewed and approved by the Ethics and Research Committee of Arnau University Hospital of Vilanova-Lliria (Valencia, Spain). All participants, including patients and healthy controls, provided written informed consent for their participation, ensuring their anonymity throughout the study.

## RESULTS

Table shows the clinical characteristics of CD patients (n = 52).

**TABLE:** Clinical characteristics of Crohn’s disease (CD) patients (n = 52)

		Mean (SD)
Age		38.8 ± 11.6
Harvey-Bradshaw Index		7.4 ± 3.6
Crohn’s Disease Activity Index		173.5 ± 116.5
		n (%)
Gender (Female)		27 (51.9)
Clinical scenarios	New patient	11 (21.2)
	Remission	14 (26.9)
	Active disease	27 (51.9)
Montreal age	A1 (< 16)	3 (5.8)
	A2 (17-40)	32 (61.5)
	A3 (> 40)	16 (30.8)
Montreal location	L1 (Ileal)	27 (51.9)
	L2 (Colonic)	4 (7.7)
	L3 (Ileocolic)	21 (40.4)
Montreal pattern	B1 (Inflammatory)	22 (42.3)
	B2 (Stenotic)	17 (32.7)
	B3 (Fistulising)	13 (25.0)

SD: standard deviation

### Anti-*A. simplex* antibodies in patients with CD and healthy subjects

**Fig. 1:**
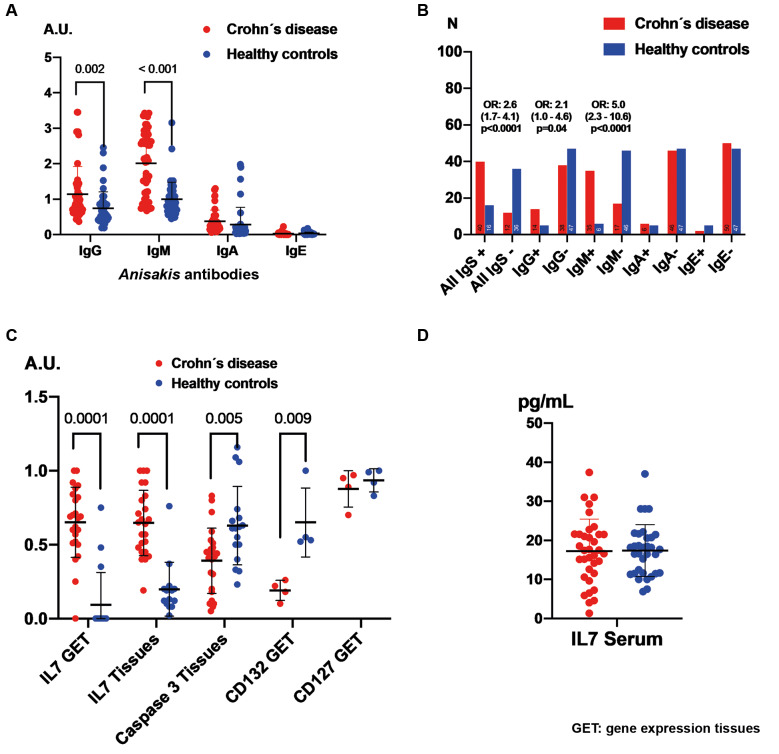
the figure presents quantitative comparisons of anti-*Anisakis simplex* antibody levels, serum interleukin 7 (IL-7), and tissue levels of IL-7, its receptor, and caspase-3 between Crohn’s disease (CD) patients and healthy controls. Panels A, C and D use the Mann-Whitney U test for continuous data, whereas Panel B displays categorical data analysed with Chi-square and Fisher’s exact tests. Means are shown with standard deviations to indicate group variability.

Fig. 1A illustrates the differences in anti-*A. simplex* antibody levels between CD patients and healthy controls, revealing significantly elevated levels of IgG and IgM in CD patients. Similarly, Fig. 1B highlights a higher prevalence of IgG and IgM positivity among CD patients. No significant variations in anti-*A. simplex* immunoglobulin levels were observed concerning sex, clinical settings, or Montreal classification.

### Values of IL-7 and its receptor caspase-3 in patients with CD and healthy subjects

The levels of IL-7 gene expression and IL-7 protein in patients with CD were significantly elevated compared to healthy controls. Conversely, caspase-3 levels were reduced in the tissues of CD patients. The expression of the IL-2 receptor subunit γ (CD132) was markedly lower in CD tissues compared to healthy subjects. However, no significant differences were observed in the expression of IL-7 receptor α (CD127) or in tissue IL-7 levels. These findings are illustrated in Fig. 1C-D.

### Assessment of IL-7 and its receptor according to positivity of anti-*A. simplex* antibodies

**Fig. 2:**
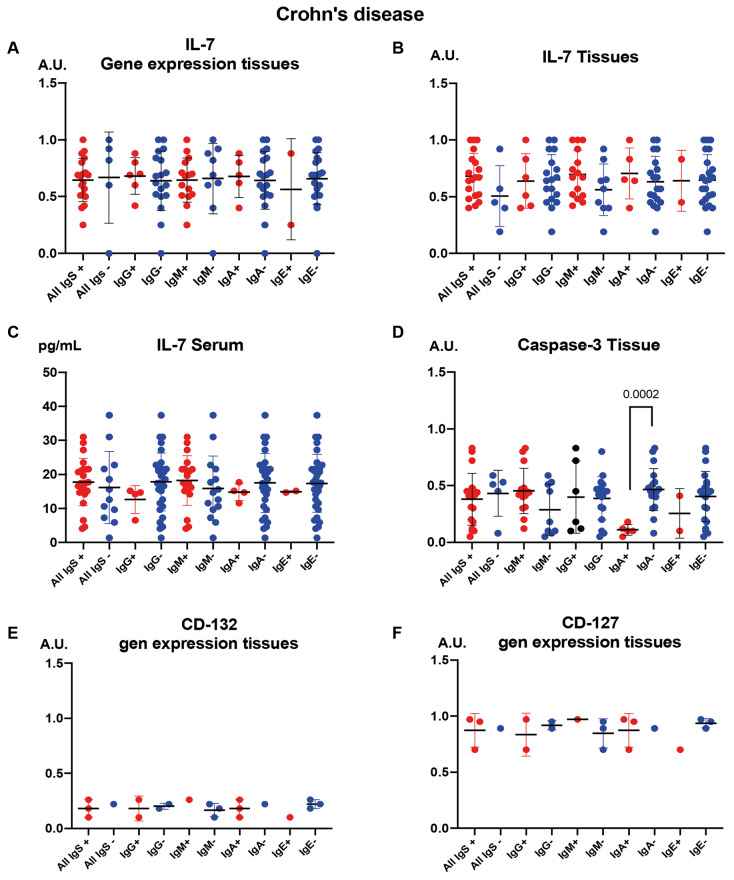
the figure stratifies the immune parameters in Crohn’s disease (CD) patients by the presence (+) or absence (-) of anti-*Anisakis simplex* antibodies. The figure is organised into panels showing: interleukin 7 (IL-7) gene expression (A); IL-7 protein levels in tissues (B); Serum IL-7 concentrations (C); Tissue caspase-3 levels (D); CD132 and CD127 receptor subunit expression (E and F). Statistical results are presented as means with double T-bars for standard deviation, with significance calculated via the Mann-Whitney U test.

Fig. 2 illustrates the expression and distribution of IL-7 and related immune markers in CD patients, stratified by the presence or absence of anti-*A. simplex* antibodies. Panel A depicts IL-7 gene expression, while Panel B shows IL-7 protein levels in tissues. Serum IL-7 concentrations are presented in Panel C, and caspase-3 levels in tissues are shown in Panel D. Panels E and F display the expression of IL-2 receptor subunits γ (CD132) and α (CD127), respectively. Notably, a significant decrease in caspase-3 levels was observed in CD patients who were positive for IgA anti-*A. simplex* antibodies (Fig. 2D).

**Fig. 3:**
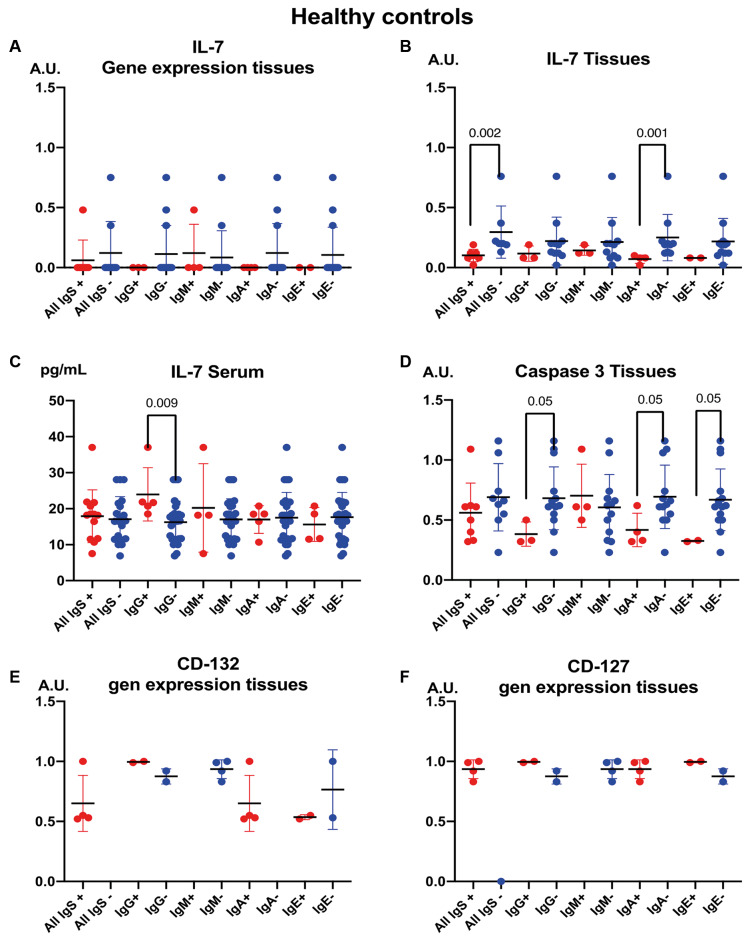
the figure focuses on healthy controls, showing how tissue interleukin 7 (IL-7) and serum IL-7 vary according to the presence (+) or absence (-) of anti-*Anisakis simplex* antibodies. Data are depicted as means in arbitrary units, with standard deviations and Mann-Whitney U statistical comparisons.

Figure 3 highlights findings in healthy individuals. Among these subjects, tissue IL-7 levels decreased in those positive for anti-*A. simplex* IgA antibodies, whereas serum IL-7 concentrations increased in individuals positive for anti-*A. simplex* IgG antibodies.

### Correlations caspase-3 and IL-7 tissues with anti-*A. simplex* antibodies

The relationship between IL-7, caspase-3, and anti-*A. simplex* antibody responses in tissues and serum can be summarised as follows.

**Fig. 4:**
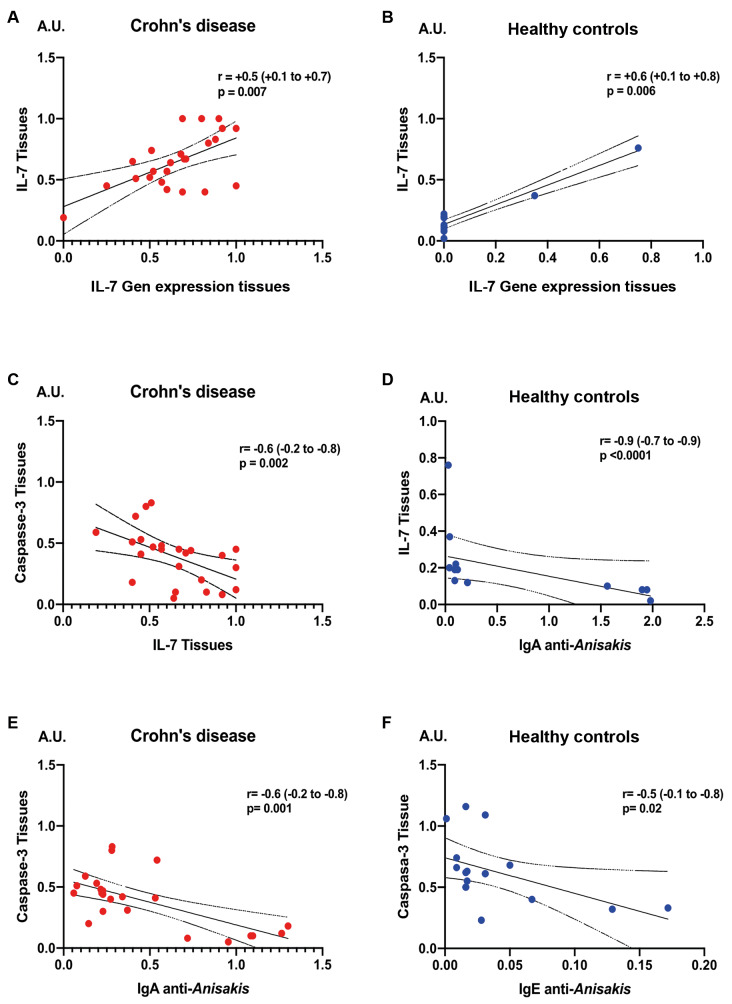
the figure analyses correlations between tissue levels of caspase-3 and interleukin 7 (IL-7) as well as serum anti-*Anisakis simplex* antibodies using Spearman’s rank correlation test, including 95% confidence intervals (CI). Both Crohn’s disease (CD) patients and controls are included for direct statistical and biological correlations.

A direct correlation existed between IL-7 gene expression and IL-7 cytokine levels in tissues for both CD patients and healthy individuals (Fig. 4A-B). In healthy subjects, tissue IL-7 levels were inversely correlated with serum IgA anti-*A. simplex* antibodies (Fig. 4D).

In CD patients, tissue caspase-3 expression was inversely related to both tissue IL-7 levels and serum IgA anti-*A. simplex* antibodies (Fig. 4C-D). Among healthy individuals, caspase-3 expression was inversely correlated with serum IgE anti-*A. simplex* antibodies (Fig. 4F).

**Fig. 5:**
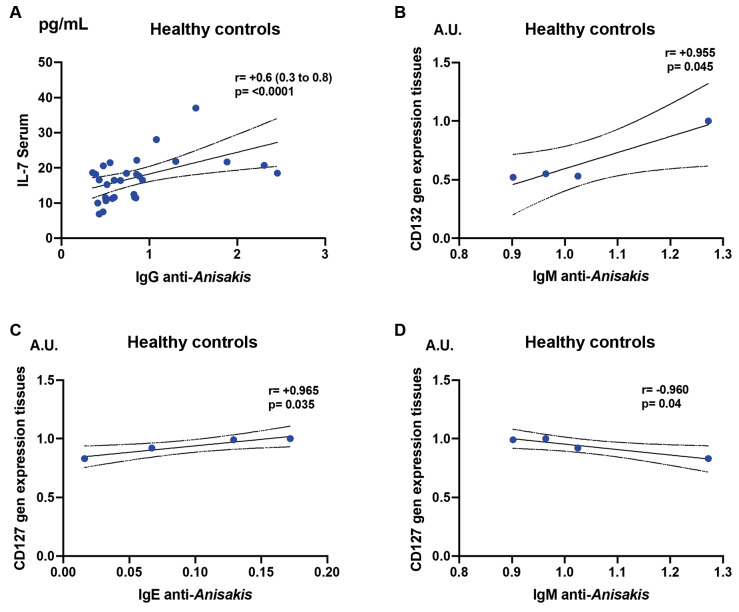
the figure explores associations between serum interleukin 7 (IL-7) levels, tissue gene expression of the IL-7 receptor, and anti-*Anisakis simplex* antibody levels using Spearman’s test. Subpanels detail positive or negative correlations with specific immunoglobulin classes, highlighting statistical significance.

In healthy individuals, serum IL-7 levels were positively correlated with serum IgG anti-*A. simplex* antibodies (Fig. 5A).

IL-2 receptor subunit γ (CD132) expression showed a positive correlation with serum IgM anti-*A. simplex* antibodies (Fig. 5B). IL-7Rα (CD127) expression was positively correlated with serum IgE anti-*A. simplex* antibodies (Fig. 5C) but negatively correlated with serum IgM anti-*A. simplex* antibodies (Fig. 5D).

All these correlations were statistically significant (p < 0.05).

### Caspase-3 protein expression and IL-7 receptor expression: IL-7 receptor α (CD127) and IL-2 receptor subunit γ (CD132) in tissues

Supplementary data (Fig. 1) shows IL-7 gene expression analysis (panel A), IL-7 protein expression (Panel B), CD127 and CD132 gene expression analysis (Panel C) and caspase-3 protein expression (Panel D). In patients with CD, tissue analysis demonstrated a significant increase in both IL-7 gene expression (p < 0.01) and IL-7 protein levels (p < 0.01) compared to healthy controls. Concurrently, caspase-3 titres in CD tissues showed a marked reduction relative to the control group (p < 0.01).

### Relationship between γδ T cells and apoptosis in CD patients compared to healthy controls

Peripheral blood analysis revealed a significant reduction in γδ T cell subsets in CD patients versus controls [Supplementary data (Fig. 2 - Panel A)], with parallel decreases observed in intestinal tissues [Supplementary data (Fig. 2 - Panel B)]. Apoptotic activity showed elevated rates in CD3+CD56+ γδ T cell subsets within peripheral blood of CD patients compared to controls [Supplementary data (Fig. 2 - Panel C)].

Notably, antibody correlations exhibited an inverse relationship between anti-*Anisakis* IgE and IgA levels and apoptosis of CD3+CD56+ γδ T cells in peripheral blood [Supplementary data (Fig. 2 - Panels D, F)]. Conversely, a positive correlation existed between CD3+CD56+ γδ T cell numbers and anti-*Anisakis* IgA titres [Supplementary data (Fig. 2 - Panel E)]. No significant associations were detected between γδ T cell subsets in intestinal tissues and *Anisakis*-specific antibodies.

Analysis of apoptosis differences in CD8+ γδ T cell subsets in peripheral blood of CD patients based on the presence or absence of anti-*Anisakis* antibodies showed a significant decrease in IgG and IgA positive patients (p = 0.013 and 0.010, respectively) (data not shown).

## DISCUSSION

Previous investigations identified a significant depletion of γδ T cell populations alongside elevated serum levels of anti-*A. simplex* immunoglobulins in CD patients.^9^ ^10^ ^12^ Recent genomic analyses further revealed diminished tissue expression of the IL-7R γ subunit (CD132), a critical component of the IL-7R heterodimer (IL-7Rα/CD127 and CD132).^10^ This downregulation of CD132 correlated with mucosal immunodeficiency, potentially disrupting IL-7-mediated survival signals for γδ T cells despite elevated tissue IL-7 expression.^10^ ^11^ The current study aimed to characterise IL-7 and its receptor dynamics in CD tissues, while investigating potential associations between these immunoregulatory pathways and anti-*A. simplex* antibody profiles, which may reflect compensatory immune responses to parasitic cofactors in disease pathogenesis.^5^ ^9^ ^12^

Our study confirmed an impaired IL-7R signalling in CD pathogenesis. As observed in previous studies, reduced gene expression of the common γ-chain receptor (CD132), a critical component of IL-7R, was observed in the intestinal tissues of patients with CD.^10^ ^11^ The relationship between IL-7R and the immune response to *A. simplex* can be inferred through the shared immunological mechanisms observed in parasitic infections and eosinophilic inflammation.^15^ ^16^ IL-7R is critical for eosinophil homeostasis in tissues.^17^ ^18^ In *A. simplex* infections, eosinophils dominate the inflammatory infiltrate during the larval penetration of the gastrointestinal mucosa. IL-7R-deficient mice showed reduced eosinophil reconstitution in the lungs, suggesting this receptor may similarly regulate eosinophilic responses to helminths.^18^ ^19^ *A. simplex* triggers a Th2-dominated response with IL-4, IL-5, and IL-13 production, which drives IgE synthesis and eosinophil recruitment. IL-7R signalling influences T cell survival and cytokine profiles, potentially modulating Th2 differentiation. Impaired IL-7R function could alter this balance, affecting parasite clearance or hypersensitivity reactions. Both *A. simplex* infection and CD show mucosal TNF-α elevation and eosinophil activation.^11^ The role of the IL-7R pathway in sustaining pathogenic T cells in CD may involve parallel mechanisms maintaining chronic inflammation in unresolved anisakiasis.

The results suggest that impaired IL-7R signalling in CD is associated with decreased expression of the common γ-chain receptor (CD132), leading to a deficiency in γδ T cells. This dysfunction may contribute to an inadequate immune response, increased susceptibility to infections, and chronic inflammation, which are critical factors in the pathogenesis of CD. These findings underscore the importance of IL-7R signalling in maintaining immune competence in affected individuals.

This deficiency disrupts the IL-7-mediated survival and maintenance of γδ T cells, contributing to their depletion in mucosal tissues. These findings suggest that restoring γδ IEL numbers or function could serve as a therapeutic strategy to prevent or mitigate CD development.^10^ ^20^

We observed compensatory IL-7 overproduction with limited efficacy. CD tissues exhibited elevated IL-7 gene expression and protein levels, likely to counteract γδ T cell loss. However, serum IL-7 levels remained unchanged, suggesting defective paracrine/autocrine signalling due to CD132 deficiency.^10^ ^21^ In CD, there was a compensatory overproduction of IL-7 in response to diminished levels of γδ T cells. However, this increase in IL-7 did not translate into effective immune restoration due to the concurrent downregulation of the common γ-chain receptor (CD132). Consequently, despite higher IL-7 levels, the signalling through its receptor remained ineffective, leading to persistent immune deficiencies and heightened vulnerability to infections and chronic inflammation within the mucosal system. This failure in compensatory mechanisms highlights a critical aspect of the dysregulated immune environment in CD.

The results showed a caspase-3 reduction and apoptosis dysregulation. Lower caspase-3 levels in CD tissues were inversely correlated with tissue IL-7 and serum levels of anti-*A. simplex* IgA, indicating impaired apoptosis regulation. This aligns with IL-7’s role in suppressing γδ T cells.^10^ ^20^ There was a notable decrease in caspase-3 expression in the tissues of CD patients, which correlated with the dysregulation of apoptosis in T cells, particularly γδ T cells. This lower caspase-3 level suggests a reduced apoptotic signal, potentially allowing for increased survival of T cells despite underlying immunodeficiency. However, this aberration in apoptosis does not rectify γδ T cell deficiency, contributing to chronic inflammation and impaired immune responses. These findings emphasise the complex interplay between reduced apoptotic signalling and maintenance of T cell homeostasis in the pathophysiology of CD. Caspase-3, a major apoptotic effector, plays a crucial role in the regulation of the immune response. Its reduction can lead to enhanced cytokine signalling and inflammation, which may contribute to the host response against *A. simplex*.^22^ Likewise, *A. simplex* produces an apoptosis-inducing protein that can trigger apoptosis in mammalian cells through two mechanisms: H_2_O_2_ production and L-lysine deprivation. Both mechanisms involve the release of cytochrome c and the activation of caspase-9.^23^ Understanding caspase-3’s role in apoptosis and immune regulation could provide insights into how *A. simplex* might evade or modulate host immune responses, a common strategy employed by parasites.^24^

Crohn’s disease patients exhibited significantly elevated levels of anti-*A. simplex* IgG and IgM antibodies compared to healthy controls. A higher prevalence of IgG and IgM positivity was also observed among CD patients. No significant differences in anti-*A. simplex* immunoglobulin levels were found when analysed by sex, clinical settings, or Montreal classification. The association between anti-*A. simplex* antibodies and immunodeficiencies have been demonstrated previously. Furthermore, our study suggests that anti-*A. simplex* antibodies could serve as markers for disease progression risk in CD. CD patients showed elevated anti-*A. simplex* IgG, IgM, and IgA antibodies, with IgA levels being linked to higher disease activity.^9^ ^12^ The deficiency of γδ T cells, which is critical for mucosal surveillance, likely creates an immunosuppressed state, permitting *A. simplex* antigen persistence and chronic antibody production.^5^ ^9^ ^12^

We observed divergent IL-7 responses in CD versus healthy subjects. In healthy subjects, serum IL-7 increased with anti-*A. simplex* IgG, suggesting a functional immune response. Conversely, CD patients exhibited reduced tissue IL-7 in IgA-positive cases, reflecting dysregulated compensatory mechanisms.^10^ ^12^ The results highlight the divergent responses of IL-7 in CD patients compared to healthy controls. In CD, there was an increase in IL-7 gene expression and protein levels in intestinal tissues, indicating an attempt to compensate for the observed deficiency of γδ T cells. In contrast, serum IL-7 levels in CD patients remain comparable to those in healthy individuals, suggesting a potential disruption in the paracrine and autocrine functions of IL-7. This discrepancy points to a pathological mechanism where despite elevated locally produced IL-7, its effectiveness is compromised due to the downregulated expression of the common γ-chain receptor (CD132). This dysfunctional signalling may contribute to persistent immune deficits in CD, highlighting the complexity of IL-7’s role in inflammation and immune regulation in affected individuals. CD patients had a higher prevalence of antibodies against *A. simplex* compared to healthy controls. This suggests an altered immune response in CD, which could be related to the divergent IL-7 responses observed in CD patients versus healthy subjects. IgA antibodies against *A. simplex* were associated with higher CD activity index. This correlation between antibody levels and disease activity might parallel potential relationships between IL-7 responses and CD severity.

Our results highlight CD132 and IL-7R signalling as potential biomarkers for CD progression and treatment resistance.^11^ Targeting this pathway could restore γδ T cell function and mitigate *A. simplex*-associated immune dysregulation.^11^ ^20^

These findings underscore a cause-effect relationship between CD132 deficiency, γδ T cell depletion, and defective mucosal immunity, which may drive both CD inflammation and susceptibility to parasitic infections like *A. simplex*.

We are aware that the study acknowledges several limitations that could impact on the validity and generalisability of its findings. The small cohort size limits statistical power, particularly for subgroup analyses, and expanding the sample size would enhance generalisability and allow for demographic stratification. While correlations between *A. simplex* infection and IL-7R expression changes are identified, causation remains unestablished. Longitudinal studies monitoring immune responses over time are needed to clarify causality and disease progression. Potential confounders like diet, concurrent infections, and treatments may skew results, necessitating detailed evaluations to isolate the role of *A. simplex*. Reliance on self-reported symptoms via the CDAI introduces bias; incorporating objective biomarkers such as faecal calprotectin or C-reactive protein could improve reliability. Additionally, mechanisms underlying *A. simplex* effects on IL-7R expression remain unexplored, requiring functional assays and in vitro studies for deeper insights. Addressing these limitations in future research would strengthen findings and understanding of immune modulation in CD.

In conclusion, the study revealed several key aspects regarding the interplay between IL-7 signalling, γδ T cell deficiency, and immune responses to *A. simplex* in CD.

Reduced gene expression of the common γ-chain receptor (CD132), a critical component of the IL-7R, was observed in CD patients’ intestinal tissues. This deficiency disrupts IL-7-mediated survival and maintenance of γδ T cells, contributing to their depletion in mucosal tissues.

Crohn’s disease tissues exhibited elevated IL-7 gene expression and protein levels, likely attempting to counteract γδ T cell loss. However, serum IL-7 levels remained unchanged, suggesting defective paracrine/autocrine signalling due to CD132 deficiency.

Lower caspase-3 levels in CD tissues correlated inversely with tissue IL-7 and serum anti-*A. simplex* IgA, indicating impaired apoptosis regulation. This aligns with IL-7’s role in suppressing γδ T cell apoptosis.

Crohn’s disease patients showed elevated anti-*A. simplex* IgG, IgM, and IgA antibodies, with IgA levels linked to higher disease activity. The deficiency in γδ T cells, critical for mucosal surveillance, likely creates an immunosuppressed state, permitting *A. simplex* antigen persistence and chronic antibody production.

In healthy subjects, serum IL-7 increased with anti-*A. simplex* IgG, suggesting a functional immune response. Conversely, CD patients exhibited reduced tissue IL-7 in IgA-positive cases, reflecting dysregulated compensatory mechanisms.

The study highlights CD132 and IL-7R signalling as potential biomarkers for CD progression and treatment resistance. Targeting this pathway could restore γδ T cell function and mitigate *A. simplex*-associated immune dysregulation.

These findings highlight the intricate interactions between cytokines, apoptosis regulators, and immune responses in both healthy individuals and those with CD.

## SUPPLEMENTARY MATERIALS

Supplementary material

## Data Availability

The contents underlying the research text are included in the manuscript.

## References

[1] Dolinger M, Torres J, Vermeire S (2024). Crohn’s disease. Lancet.

[2] Andreu-Ballester JC, Amigo-Garcia V, Catalan-Serra I, Gil-Borras R, Ballester F, Almela-Quilis A (2011). Deficit of gammadelta T lymphocytes in the peripheral blood of patients with Crohn’s disease. Dig Dis Sci.

[3] Gilliland A, Chan JJ, De Wolfe TJ, Yang H, Vallance BA (2024). Pathobionts in inflammatory bowel disease: origins, underlying mechanisms, and implications for clinical care. Gastroenterology.

[4] del Pozo V, Arrieta I, Tunon T, Cortegano I, Gomez B, Cardaba B (1999). Immunopathogenesis of human gastrointestinal infection by *Anisakis simplex*. J Allergy Clin Immunol.

[5] Gutierrez R, Cuellar C (2002). Immunoglobulins anti-*Anisakis simplex* in patients with gastrointestinal diseases. J Helminthol.

[6] Baron L, Branca G, Trombetta C, Punzo E, Quarto F, Speciale G (2014). Intestinal anisakidosis: histopathological findings and differential diagnosis. Pathol Res Pract.

[7] de la Hoz-Martin MP, Gonzalez-Fernandez J, Andreu-Ballester JC, Hoivik ML, Ricanek P, Bruland T (2025). Prevalence of anti-*anisakis simplex* antibodies in a cohort of patients with inflammatory bowel disease in Norway. Pathogens.

[8] Aibinu IE, Smooker PM, Lopata AL (2019). *Anisakis* nematodes in fish and shellfish- from infection to allergies. Int J Parasitol Parasites Wildl.

[9] Benet-Campos C, Cuellar C, Garcia-Ballesteros C, Zamora V, Gil-Borras R, Catalan-Serra I (2017). Determination of anti-*Anisakis simplex* antibodies and relationship with alphabeta and gammadelta lymphocyte subpopulations in patients with Crohn’s disease. Dig Dis Sci.

[10] Andreu-Ballester JC, Hurtado-Marcos C, Garcia-Ballesteros C, Perez-Griera J, Izquierdo F, Ollero D (2025). Decreased gene expression of interleukin 2 receptor subunit gamma (CD132) in tissues of patients with Crohn’s disease. World J Gastroenterol.

[11] Belarif L, Danger R, Kermarrec L, Nerriere-Daguin V, Pengam S, Durand T (2019). IL-7 receptor influences anti-TNF responsiveness and T cell gut homing in inflammatory bowel disease. J Clin Invest.

[12] Guillen-Bueno R, Gutierrez-Ramos R, Perteguer-Prieto MJ, Olveira-Martin A, Fernandez-Blanco I, Pozuelo-Garcia A (1999). Anti-*anisakis* antibodies in the clinical course of Crohn’s disease. Digestion.

[13] Lennard-Jones JE (1989). Classification of inflammatory bowel disease. Scand J Gastroenterol Suppl.

[14] Papay P, Ignjatovic A, Karmiris K, Amarante H, Milheller P, Feagan B (2013). Optimising monitoring in the management of Crohn’s disease: a physician’s perspective. J Crohns Colitis.

[15] Huang L, Appleton JA (2016). Eosinophils in helminth infection: defenders and dupes. Trends Parasitol.

[16] Yasuda K, Kuroda E (2019). Role of eosinophils in protective immunity against secondary nematode infections. Immunol Med.

[17] Jacobs SR, Michalek RD, Rathmell JC (2010). IL-7 is essential for homeostatic control of T cell metabolism in vivo. J Immunol.

[18] Cool T, Worthington A, Poscablo D, Hussaini A, Forsberg EC (2020). Interleukin 7 receptor is required for myeloid cell homeostasis and reconstitution by hematopoietic stem cells. Exp Hematol.

[19] Maki K, Sunaga S, Komagata Y, Kodaira Y, Mabuchi A, Karasuyama H (1996). Interleukin 7 receptor-deficient mice lack gammadelta T cells. Proc Natl Acad Sci USA.

[20] Xu W, Golovchenko NB, Martinez-Vargas IU, Fong A, Rout P, Achi S (2025). Dysregulation of gammadelta intraepithelial lymphocytes precedes Crohn’s disease-like ileitis. Sci Immunol.

[21] Ishiguro Y, Kanazawa H, Yamagata K, Sakuraba H, Munakata A (2001). Existence of variant gamma delta T cells in Crohn’s disease. Digestion.

[22] Wu D, Wang Z, Zhang J, Robinson AG, Lyu B, Chen Z (2022). Apoptotic caspase inhibits innate immune signaling by cleaving NF-κBs in both Mammals and Flies. Cell Death Dis.

[23] Murakawa M, Jung SK, Iijima K, Yonehara S (2001). Apoptosis-inducing protein, AIP, from parasite-infected fish induces apoptosis in mammalian cells by two different molecular mechanisms. Cell Death Differ.

[24] Chulanetra M, Chaicumpa W (2021). Revisiting the mechanisms of immune evasion employed by human parasites. Front Cell Infect Microbiol.

