# ERRATUM: Immune dysregulation, apoptosis impairment, and enhanced seroreactivity to Anisakis simplex in Crohn’s disease: interplay of IL-7/IL-7R signalling and CD132 deficiency

**DOI:** 10.1590/0074-02760250129er

**Published:** 2026-11-13

In the article “**Immune dysregulation, apoptosis impairment, and enhanced seroreactivity to Anisakis simplex in Crohn’s disease: interplay of IL-7/IL-7R signalling and CD132 deficiency**”, DOI number: 10.1590/0074-02760250129, published in *Mem Inst Oswaldo Cruz*, Rio de Janeiro, Vol. 121: e250129, 2026, on page 1:

**Where it reads**:

Carmen Cuéllar^1,2^/^+^, Carolina Hurtado-Marcos^3^, Elizabeth Valdivieso^3^, Lucianna Vaccaro^3^, Juan González-Fernández^1,2^, Ana Isabel Jiménez^4^, Jaume Pérez-Griera^5^, Salvador Benlloch^6,7^, Cirilo Amorós^6^, Rafael Gil-Borrás^7^, Rosa Sorando-Serra^8^, María José Cano-Cano^8^, Juan Carlos Andreu-Ballester^2,9^

^1^Complutense University of Madrid, Department of Microbiology and Parasitology, Madrid, Spain ^2^Complutense University of Madrid, Parasitic Immunobiology and Immunomodulation Research Group, Madrid, Spain ^3^CEU Universities, Universidad CEU San Pablo, Urbanización Montepríncipe, Boadilla del Monte, Spain ^4^Arnau de Vilanova Hospital, Biopathology Department, Valencia, Spain ^5^University Clinical Hospital, Laboratory Department, Valencia, Spain ^6^Arnau de Vilanova Hospital, Digestive Departament, Valencia, Spain ^7^CEU Universities, Cardenal Herrera, Valencia, Spain ^8^Arnau de Vilanova Hospital, Emergency Department, Valencia, Spain ^9^Foundation for the Promotion of Health and Biomedical Research in the Valencian Region, Benimaclet, Valencia, Spain

**It should read**:

Carmen Cuéllar^1,2^/^+^, Carolina Hurtado-Marcos^3^, Elizabeth Valdivieso^3^, Lucianna Vaccaro^3^, Juan González-Fernández^1,2^, Ana Isabel Jiménez^4^, Jaume Pérez-Griera^5^, Salvador Benlloch^6,7,10^, Cirilo Amorós^6^, Rafael Gil-Borrás^7^, Rosa Sorando-Serra^8^, María José Cano-Cano^8^, Juan Carlos Andreu-Ballester^2,9^

^1^Complutense University of Madrid, Department of Microbiology and Parasitology, Madrid, Spain ^2^Complutense University of Madrid, Parasitic Immunobiology and Immunomodulation Research Group, Madrid, Spain ^3^CEU Universities, Universidad CEU San Pablo, Urbanización Montepríncipe, Boadilla del Monte, Spain ^4^Arnau de Vilanova Hospital, Biopathology Department, Valencia, Spain ^5^University Clinical Hospital, Laboratory Department, Valencia, Spain ^6^Arnau de Vilanova Hospital, Digestive Departament, Valencia, Spain ^7^CEU Universities, Cardenal Herrera, Valencia, Spain ^8^Arnau de Vilanova Hospital, Emergency Department, Valencia, Spain ^9^Foundation for the Promotion of Health and Biomedical Research in the Valencian Region, Benimaclet, Valencia, Spain ^10^CIBERehd, Instituto de Salud Carlos III, Madrid, Spain

