# Supplementary material for: Immune dysregulation, apoptosis impairment, and enhanced seroreactivity to Anisakis simplex in Crohn’s disease: interplay of IL-7/IL-7R signalling and CD132 deficiency
Source: Mem Inst Oswaldo Cruz. 2026 Apr 10;121:e250129. doi: 10.1590/0074-02760250129 (PMC13078791; doi:10.1590/0074-02760250129)
Supplement: Supplementary material [file 1678-8060-mioc-121-e250129-s1.pdf]

TABLE A

The primers sequences of interleukin 7 (IL-7) and IL-7 receptor for quantitative polymerase chain reaction (qPCR)

	Foward	Reverse
IL7	GAG TGA CTA TGG GCG GTG AGA G	GAT GCT ACT GGC AAC AGA ACA AGG
CD127 (IL7Rα)	CACCCAAGTCAATGCCTTTT	TGAGCATTCAGTAGCCATGC
CD132 (IL2Rγ)	TATGTGCTCCTGCTCCCTCT	ACCCCCACACTCTGTCTGTC
GADPH	TGAGGCCGGTGCTGAGTATGTCG	CCACAGTCTTCTGGGTGGCAGTG

TABLE B

The antibodies used in Western-Blot

	Antibody	Dilution	Company	Reference
Caspase-3	Anti-caspase-3	1:100 (WB)	AbCam	ab4051
IL-7	Anti-IL7	1:100 (WB)	AbCAm	Ab9732
Secondary antibody	Anti-rabbit Ig/HRP	1:1000 (WB)	SIGMA	PO545
Actin	Anti-actin	1:500 (WB)	SIGMA	A5441

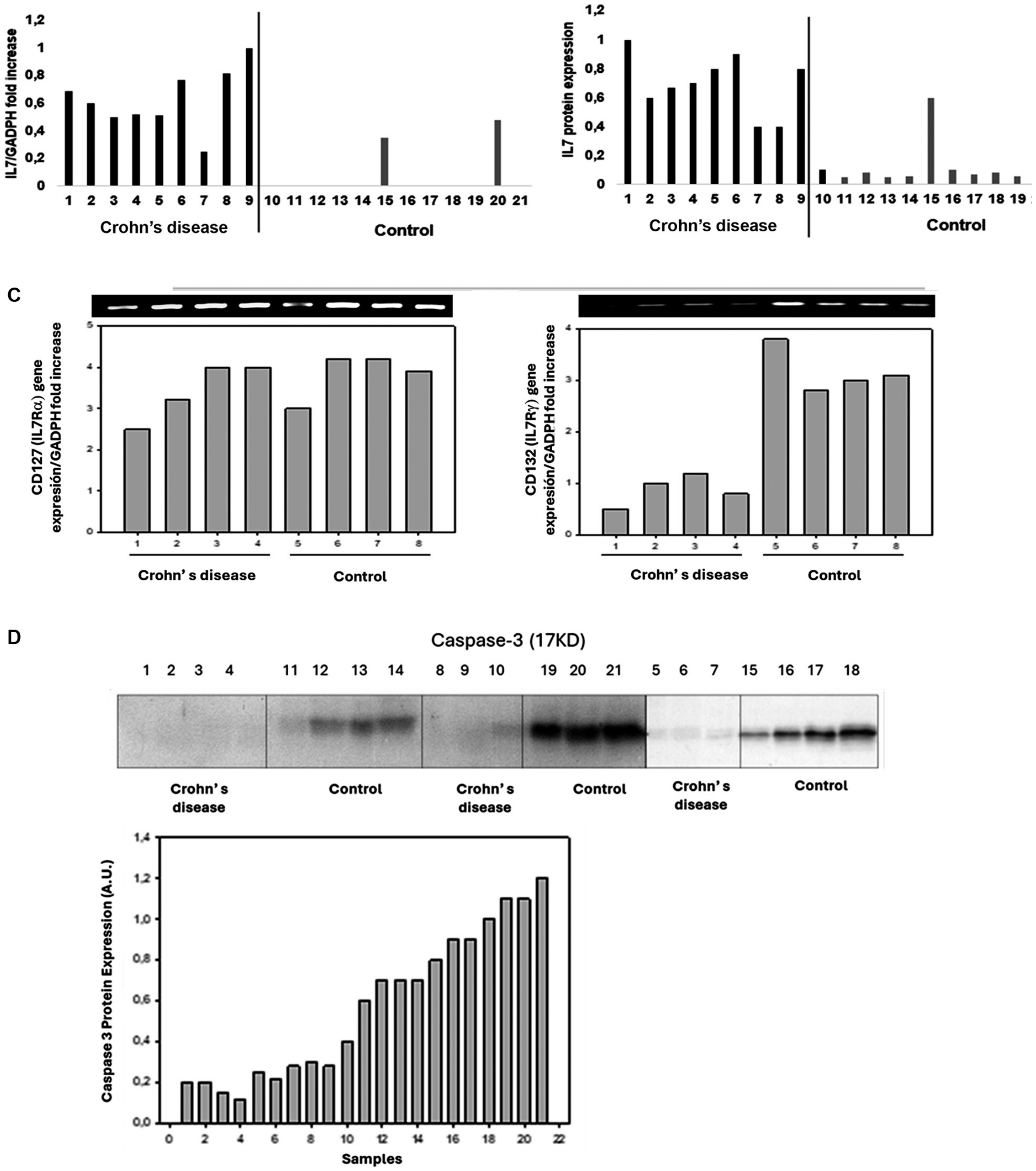

E

Characteristics of patients and controls					
Nº	Gender	Age	Case/Control	Antivity	Montreal
1	Female	53	Crohn	Active	A3-L1-B1
2	Male	42	Crohn	Remission	A3-L1-B1
3	Male	42	Crohn	Remission	A3-L3-B2
4	Male	22	Crohn	Remission	A2-L1-B1
5	Female	67	Control		
6	Female	59	Control		
7	Male	49	Control		
8	Female	49	Control		

Fig. 1: (Panel A) Interleukin 7 (IL-7) gene expression analysis. The relative expression of the IL-7 gene was quantified using reverse transcription quantitative polymerase chain reaction (RT-qPCR). Gene expression levels were normalised against glyceraldehyde-3-phosphate dehydrogenase (GAPDH), which served as the reference standard. (Panel B) IL-7 protein expression. Western blot analysis was conducted using protein extracts from intestinal biopsies to evaluate IL-7 protein levels. Anti-actin (1:1000) was used as the loading control (data not shown). Quantification of the visualised protein fragments was performed using densitometry with ImageJ software (National Institutes of Health, Bethesda, MD, USA). (Panel C) CD127 and CD132 gene expression analysis. The expression levels of CD127 and CD132 genes were assessed through RT-qPCR. Similar to Panel A, GAPDH was used as the reference gene for normalisation. (Panel D) Caspase-3 protein expression. Western blot analysis was employed to measure caspase-3 protein levels in intestinal biopsy samples. Anti-actin (1:1000) served as the loading control (data not shown). Densitometric quantification of the protein bands was carried out using ImageJ software. (Panel E) Characteristics of Crohn's disease (CD) patients and healthy subjects. This panel provides a comparative summary of clinical and demographic characteristics between patients diagnosed with CD and healthy control subjects.

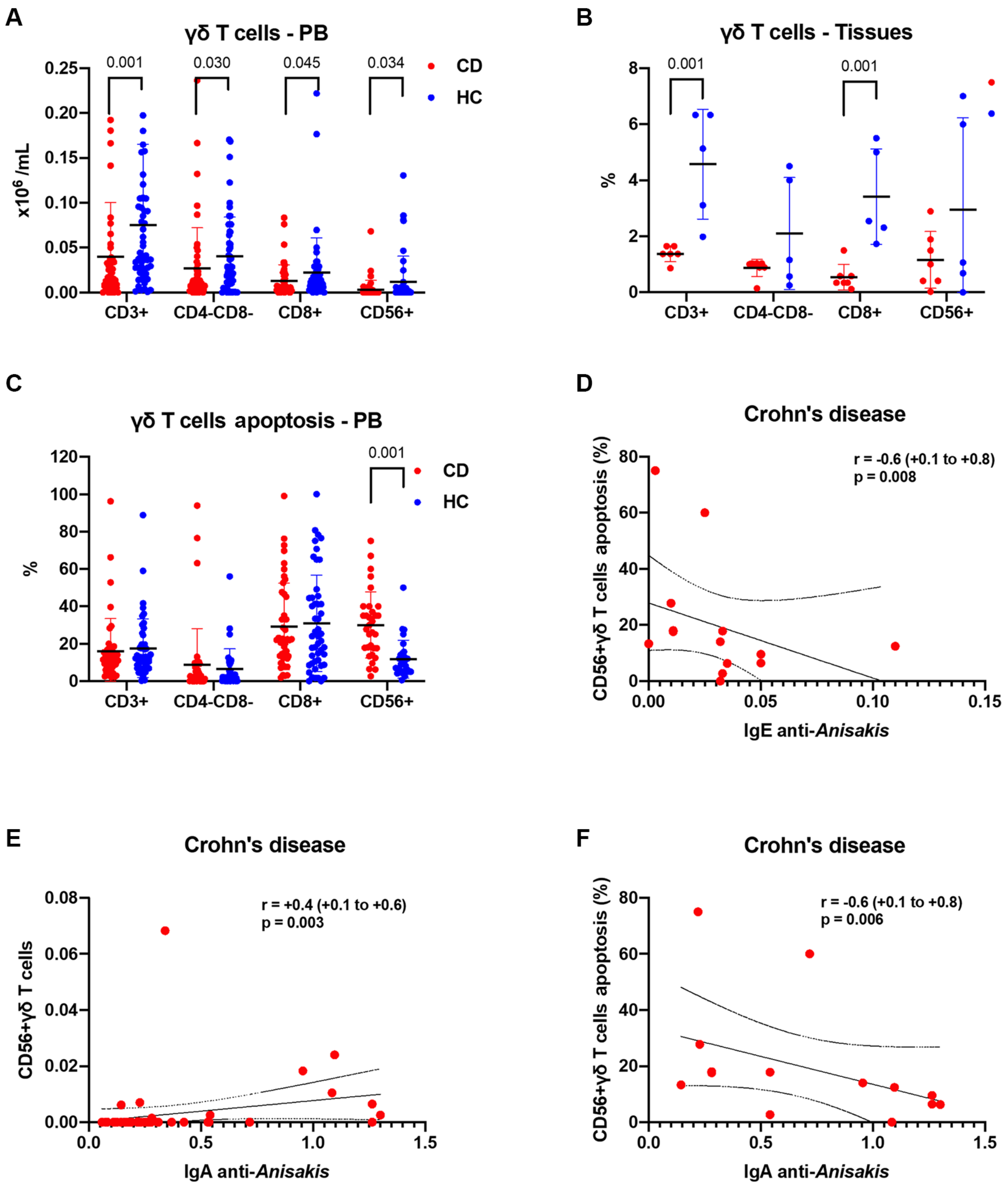

Fig. 2: analysis of the relationship between γδ T cells and their apoptosis in patients with Crohn's disease (CD) compared to healthy controls (HC). (Panel A) Differences in the number of γδ T cell subsets in peripheral blood (PB) between CD patients and HC. (Panel B) Differences in the number of γδ T cell subsets within tissue samples from CD patients and HC. (Panel C) Differences in apoptosis rates of γδ T cell subsets in peripheral blood between the two groups. Statistical comparisons for Panels A, B, and C were conducted using the Mann-Whitney U test. (Panels D, E, and F) Significant correlations between γδ T cell subsets and anti-*Anisakis simplex* antibodies. Spearman's rank correlation test was employed to calculate the correlation coefficients (r) along with their 95% confidence intervals (CI).
